# Effects of Exercise on Arterial Stiffness: Mechanistic Insights into Peripheral, Central, and Systemic Vascular Health in Young Men

**DOI:** 10.3390/metabo15030166

**Published:** 2025-03-01

**Authors:** Yongsheng Lan, Ruisi Wu, Yujuan Feng, Teng Keen Khong, Cunhan Wang, Ashril Yusof, Guangwei Che

**Affiliations:** 1 Faculty of Sports and Exercise Science, Universiti Malaya, Kuala Lumpur 50603, Malaysia; lanys@ccsfu.edu.cn (Y.L.); khongtk@um.edu.my (T.K.K.); 2 College of Physical Education, Changchun Normal University, 677 North Changji Road, Changchun 130032, China; qx202314005@stu.ccsfu.edu.cn (R.W.); qx202314008@stu.ccsfu.edu.cn (C.W.); 3 General Education Section, Shandong University of Art and Design, No. 23 Qianfushan East Road, Lixia District, Jinan 250399, China; fengyujuan@stu.sdada.edu.cn; 4 School of Rehabilitation Medicine, Shandong University of Traditional Chinese Medicine, No. 4655, University Road, University Science and Technology Park, Changqing District, Jinan 250355, China

**Keywords:** exercise, peripheral arterial stiffness, central arterial stiffness, systemic arterial stiffness, young men

## Abstract

Background/Objectives: Arterial stiffness, a critical predictor of cardiovascular events, varies regionally across peripheral, central, and systemic arteries, necessitating targeted exercise interventions for young men. However, research on the effects of exercise on arterial stiffness in these regions among young men remains limited. This review aims to (i) examine the effects of exercise on arterial stiffness in young men across these regions, and (ii) investigate the underlying mechanisms involved. Methods: Database searches on PubMed, ScienceDirect, Web of Science, and Scopus were conducted up to July 2024. The keywords were: exercise, men/male, and arterial stiffness. Inclusion criteria were studies involving young men, supervised exercise, and arterial stiffness measures. Thirty-five papers were categorized into groups based on peripheral, central and systemic arterial stiffness. Results: Peripheral arterial stiffness: continuous aerobic cycling (light to high intensity), interval aerobic cycling (moderate to high intensity), and 30-s stretching exercises demonstrated positive effects, likely due to short-term changes in sympathetic nervous system activity, nitric oxide availability, and vascular tone. Central arterial stiffness: chronic high-intensity continuous and interval aerobic cycling exercises promoted vascular remodeling, including elastin preservation and collagen regulation. For systemic arterial stiffness, continuous and interval aerobic cycling and light-intensity squats with whole-body vibration exercises improve endothelial function, smooth muscle relaxation, and vascular remodeling. Conclusions: Tailored exercise intervention can effectively reduce arterial stiffness across peripheral, central and systemic regions in young men. Improvements in peripheral stiffness are linked to short-term metabolic shifts, central stiffness responds to long-term remodeling, while systemic arterial stiffness involves both short- and long-term metabolic adaptations.

## 1. Introduction

Cardiovascular diseases (CVDs) remain the leading cause of mortality worldwide, with death rates increasing by 15% over the past two decades [1]. Arterial stiffness, a measure of arterial elasticity, has been recognized as a significant independent predictor of CVD [2]. Notably, arterial stiffness increases with age, with the risk of developing high arterial stiffness increasing by 14.6% with each added year of age [3]. This highlights the importance of addressing arterial stiffness early in life, even in individuals without overt cardiovascular risk factors [4]. Moreover, young men exhibit a higher prevalence of elevated arterial stiffness compared to young women (23.6% vs. 10.6%) [5,6]. This disparity is likely to be attributable to the protective vascular effects of estrogen in women [7] and the higher levels of low-density lipoprotein cholesterol in men [8]. These findings emphasize the need for effective strategies to reduce arterial stiffness specifically in young men.

There are several categories of arterial stiffness: peripheral, central, and systemic [9,10]. Peripheral arterial stiffness refers to stiffness in muscular arteries, such as the radial, femoral, and brachial arteries [11]. It is commonly assessed using foot-to-brachial pulse wave velocity (faPWV) and is closely linked to peripheral arterial disease [12] and chronic kidney disease [13]. Peripheral arterial stiffness is primarily influenced by blood pressure and sympathetic nervous system activity [14]. Metabolic pathways affecting peripheral arterial stiffness include vascular contraction mediated by sympathetic nervous system overactivation [15], the formation of advanced glycation end-products due to hyperglycemia [16], and inflammation-driven dyslipidemia [17]. Central arterial stiffness pertains to elasticity in large arteries, such as the aorta, and is typically measured using carotid-femoral pulse wave velocity (cfPWV). It is a well-established predictor of cardiovascular events, including heart failure [18] and isolated systolic hypertension [19]. Unlike peripheral arterial stiffness, central arterial stiffness is predominantly influenced by structural changes in the arterial wall, such as elastin degradation and collagen accumulation, which occur with aging and chronic disease [20]. Key metabolic pathways associated with central stiffness include elastin degradation and collagen cross-linking due to pulsatile pressure [21], endothelial dysfunction caused by oxidative stress [22], and vascular calcification associated with aging [23]. Systemic arterial stiffness provides a comprehensive measure of overall arterial health and is often evaluated using an index such as the cardio-ankle vascular index (CAVI) [24]. Exercise has been shown to significantly reduce arterial stiffness in older adults [25,26,27]. However, physiological differences between young and older individuals, such as increased arterial stiffness resulting from age-related reductions in elastin and increased collagen deposition [28], as well as metabolic dysfunction characterized by impaired insulin sensitivity and heightened systemic inflammation [29], necessitate tailored exercise prescriptions for young adults to prevent CVDs. Despite these findings, the effects of exercise on arterial stiffness in young men across peripheral, central, and systemic regions remain underexplored. Additionally, the metabolic pathways underlying these effects in this population are poorly understood, emphasizing the need for further research.

This review aims to synthesize the current evidence on the effectiveness of exercise interventions in improving peripheral, central and systemic arterial stiffness in young men, with a specific focus on the underlying metabolic mechanisms. The findings aim to provide targeted exercise guidelines for young men to prevent specific CVDs and offer mechanistic insights into the effects of exercise on arterial stiffness among these three regions.

## 2. Methods

### 2.1. Search Strategy

Literature searches were conducted through 4 databases: Web of Science, Scopus, PubMed, and ScienceDirect, by one team member. The search was conducted from the date of inception until July 2024. Table 1 shows the search strategy using the Web of Science as an example. A similar search strategy was used on the other databases. The reference lists of included studies and cited articles were cross-searched for other potentially eligible studies using the same inclusion criteria. This research was registered in PROSPERO on 16 January 2023 (CRD42023387815).

### 2.2. Study Selection

**Figure 1:**
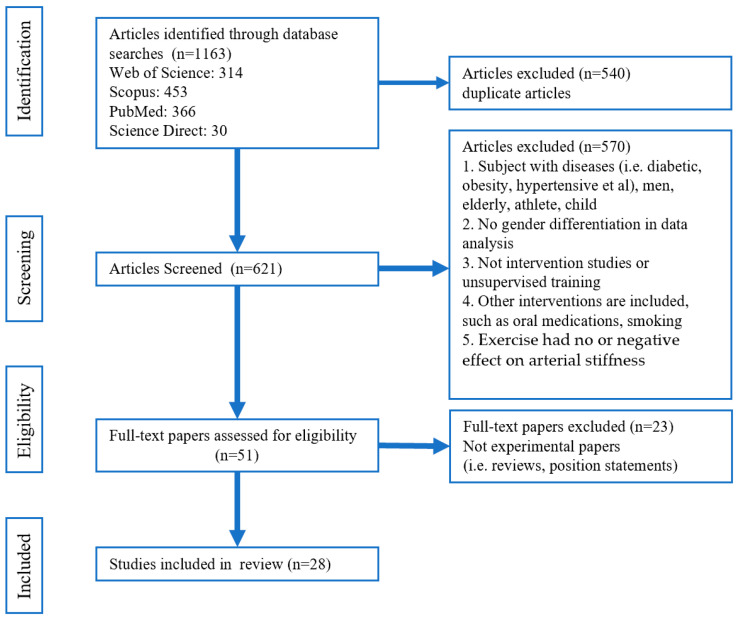
PRISMA flowchart for study selection.

Figure 1 displays the flow chart of the search. The number of articles in the databases by search strategy was 314 in Web of Science, 453 in Scopus, 366 in PubMed, and 30 in ScienceDirect, for a total of 1163 articles. All identified papers were entered by two team members into EndNote 9 to ascertain duplicates and excluded 602 duplicate articles. Furthermore, articles were further excluded based on the following principles: non-experimental articles (reviews, conference proceedings, etc.); sample characteristics do not meet the study criteria, e.g., elderly, women, athletes, people with diseases; data analysis is not gender-specific; experiment included other interventions (diet, medication, etc.); exercise had no or negative effect on arterial stiffness. In addition, only articles with male participants aged 18–25 years old were included. Finally, a total of 28 articles were included in the review.

### 2.3. Data Extraction and Quality Assessment

Relevant data were extracted from each article independently by three team members. The data consisted of exercise prescription information (mode, time, frequency, intensity, duration) and outcome findings for arterial stiffness.

Discrepancies regarding the inclusion of any articles were discussed with a third reviewer and resolved when a consensus was reached. The study quality were assessed using the Hawker et al.’s nine-item checklist (Appendix A Table A1, Table A2 and Table A3).

The extracted papers were categorized into peripheral (Table 2), central (Table 3) and systemic arterial stiffness (Table 4) groups. Descriptive summaries of the studies were included in the study.

## 3. Results and Discussion

### 3.1. Classification of Exercise Intensity and Arterial Stiffness

#### 3.1.1. Exercise Intensity Classification

The intensity of exercise in this review is defined by percentage of maximum heart rate (HRmax), peak oxygen uptake (VO_2_peak), heart rate reserve (HRR), maximum oxygen consumption (VO_2_max), peak power output, and repetition maximum (RM). Light intensity was recognized as equal to or less than 63% HRmax, 45% VO_2_peak, 30% HRR, 51% VO_2_max, 40% PPO, and 67% 1RM. Moderate intensity was recognized as 64–76% HRmax, 46–63% VO_2_peak, 31–50% HRR, 52–67% VO_2_max, 41–60% PPO, and 67–85% 1RM. The high intensity was recognized as more than 76% HRmax, 63% VO_2_peak, 50% HRR, 67% VO_2_max, 60% PPO, and 85% 1RM [55,56,57,58,59,60,61].

#### 3.1.2. Arterial Stiffness Classification

Indicators that reflect the stiffness of arteries in the limbs and smaller vessels, such as faPWV, pPWV (peripheral pulse wave velocity), fdPWV (femoral–distal pulse wave velocity), ulPWV (upper limb pulse wave velocity), brPWV (brachial artery pulse wave velocity), bPWV (brachial pulse wave velocity), and llPWV (lower limb pulse wave velocity), are considered markers of peripheral arterial stiffness. Indicators reflecting the stiffness of large arteries in the central circulatory system include cfPWV, cPWV (central pulse wave velocity), aPWV (aortic pulse wave velocity), and the β Stiffness Index, which is considered a marker of central arterial stiffness. Indicators reflecting overall arterial stiffness across the entire vascular system, including CAVI and baPWV (brachial-ankle pulse wave velocity), are regarded as markers of systemic arterial stiffness.

### 3.2. Effects of Exercise on Peripheral Arterial Stiffness and Metabolic Pathways

Peripheral arterial stiffness primarily affects muscular arteries, such as the femoral, radial, and brachial arteries, which play a crucial role in regulating blood flow to specific tissues. These smaller arteries are particularly susceptible to localized vascular conditions, like peripheral artery disease (PAD) [62,63], which is becoming increasingly prevalent among young adults. Recent studies indicate a 7.38% increase in the global prevalence of PAD in young adults [64], with young men facing a 13% higher risk of all-cause mortality compared to women diagnosed with PAD [65]. Given the growing concern regarding the impact of PAD on young men, addressing peripheral arterial stiffness from early in life is crucial for mitigating future cardiovascular risks.

Three types of exercise, continuous aerobic exercise, interval aerobic training, and stretching exercises, have been shown to positively influence peripheral arterial stiffness in young men (Table 2). For continuous aerobic exercise, light-intensity cycling at a heart rate (HR) of 82 bpm for 5 min demonstrated a reduction in faPWV [30]. Moderate-intensity cycling at 50% maximum aerobic work capacity [33] reduced fdPWV. High-intensity cycling, performed at either maximal aerobic capacity or 70% HRR, significantly decreased peripheral arterial stiffness [34,66]. For interval aerobic training, moderate-intensity significantly reduced pPWV. Rakobowchuk et al observed a reduction in pPWV after four Wingate tests [35]. Similarly, Tordi et al reported reductions in llPWV with an average exercise HR of 144 bpm [36]. High-intensity interval aerobic training, such as cycling at 100% maximum aerobic work capacity [33] and at 65% PPO [37], also led to significant reductions in peripheral arterial stiffness measures. For stretching exercises, maximal exertion systemic static stretching protocols resulted in notable reductions in peripheral arterial stiffness. Yamato et al reported significant decreases in faPWV following stretching regimens involving 40 min of 30-s holds with 10-s relaxations, as well as six 30-s static stretches with 10-s recoveries [38,39]. Higaki et al further corroborated these findings, observing reductions in faPWV after stretching exercises performed to the point of minimal discomfort [40].

**Figure 2:**
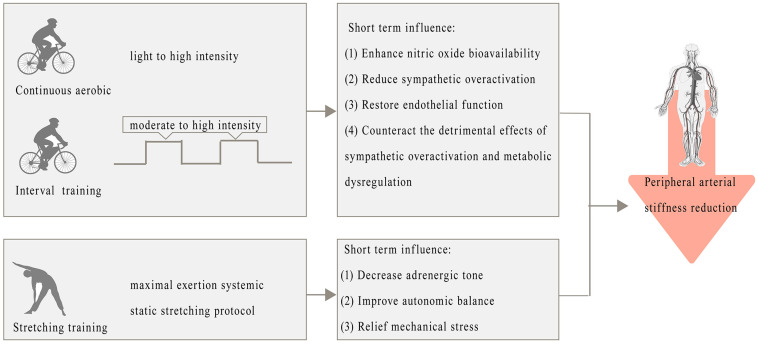
Positive effects of exercise on peripheral arterial stiffness and metabolic pathways.

Exercise has a profound impact on peripheral arterial stiffness, particularly in response to short-term fluctuations. The metabolic pathways driving these changes reflect a complex interaction between endothelial function, vascular tone, and sympathetic modulation. During exercise, acute increases in HR and blood pressure [67] trigger adaptive responses that improve vascular health. For example, moderate-intensity continuous and interval aerobic training enhances nitric oxide bioavailability and reduces sympathetic overactivation, helping to restore endothelial function and subsequently reduce vascular stiffness [68]. The immediate effects of exercise are thought to result from temporary improvements in vasodilation and shifts in vascular tone, driven by decreased adrenergic tone and improved autonomic balance [69]. In particular, stretching exercises, even when performed to the point of minimal discomfort, have demonstrated significant reductions in faPWV, underscoring the positive impact of mechanical stress relief on vascular function [70]. These findings suggest that exercise, especially when performed at moderate to high intensities, not only directly influences peripheral arterial stiffness but also mitigates the detrimental effects of sympathetic overactivation and metabolic dysregulation (Figure 2).

### 3.3. Effects of Exercise on Central Arterial Stiffness and Metabolic Pathways

Central arterial stiffness, which refers to the stiffness of large arteries, such as the aorta and carotid arteries, is a major independent determinant of cardiovascular disease risk. It is closely associated with several cardiovascular conditions, including isolated systolic hypertension, heart failure, and atherosclerosis [71]. Normally, central arteries are less stiff than peripheral arteries. However, as individuals age or develop cardiovascular disease, central stiffness can equal or exceed peripheral stiffness, leading to impedance mismatching [72]. It is noteworthy that young men exhibit higher cfPWV than young women by approximately 0.35 m/s [73], highlighting the need for early interventions to address central arterial health in this population.

Two exercise types, continuous and interval aerobic training, positively influence central arterial stiffness in young men (Table 3). For continuous aerobic exercise, prolonged high-intensity cycling has been associated with decreased central arterial stiffness (65% VO_2_peak for 6 days [32] and 60–70% VO_2_max for 8 weeks [42]). In contrast, acute exercise indicated no significant changes in central arterial stiffness following high intensity, continuous aerobic cycling [66], 50% maximum aerobic work capacity [33] and 60% PPO for 30 min [44], treadmill exercise at 90% HRmax [74], and 70% HRR [34] for 45 min. For interval aerobic training, positive effects on central arterial stiffness have been observed: (i) regular interval training (6 weeks); (ii) submaximal intensity (60 and 65% PPO), and (iii) intervals exceeding 4 min (4–15 min). As evidenced, 6-week Wingate tests at 65% VO_2_peak, interspersed with 4.5 min recovery [43], 6-week cycling exercise at 170% VO_2_max, 6–7 sets of 20 s exercise, 10 s rest [42], cycling exercise at 65% PPO, 2 bouts of 15 min separated by 20-min intervals [37], and cycling exercise at 60% PPO, 4-min intervals [44] all showed a reduction in cfPWV. However, no positive changes were found with interval aerobic training at maximal high intensity (100% maximum aerobic work capacity) and intervals less than 2 min (0.5–2 min) [33,75,76]. Therefore, the intensity and duration of interval training are important factors affecting central arterial stiffness.

**Figure 3:**
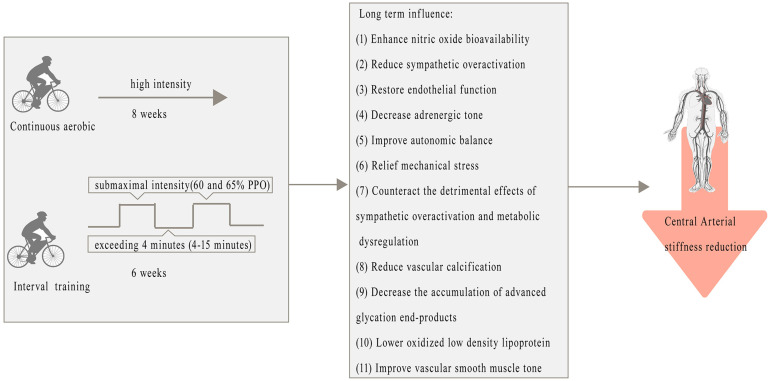
Positive Effects of exercise on central arterial stiffness and metabolic pathways.

The improvement of central arterial stiffness appears to be more challenging than improving peripheral arterial stiffness through exercise. This may be due to the following: (i) central arterial stiffness is mainly influenced by age [77]; (ii) central arteries are subject to higher pulsatile forces and have a more complex elastic structure [78], making them less responsive to the same training stimuli that may benefit peripheral arteries. In this review, regular exercise has consistently demonstrated beneficial effects on central arterial stiffness, particularly through structural and functional adaptations in the vasculature. Unlike the more transient improvements observed in peripheral arterial stiffness, central arterial stiffness responds primarily to chronic exercise due to its dependency on structural remodeling processes, such as elastin preservation and collagen regulation [79]. Continuous and interval aerobic training, when performed at moderate intensities (e.g., 60–65% PPO or VO_2_peak) and over extended durations (e.g., 6 weeks), effectively reduces cfPWV, likely by mitigating hemodynamic stress and improving endothelial function [80]. These protocols promote favorable adaptations, such as reduced vascular calcification, decreased accumulation of advanced glycation end-products (AGEs), lower oxidized low-density lipoprotein [81], and improved vascular smooth muscle tone [82]. However, high-intensity exercise combined with short recovery intervals appears less effective or even counterproductive, potentially due to excessive hemodynamic strain and heightened oxidative stress, which impair vascular repair processes [83]. The unique responsiveness of central arteries to regular, sustained exercise highlights the importance of optimizing exercise prescriptions to balance intensity, duration, and recovery (Figure 3).

### 3.4. Effects of Exercise on Systemic Arterial Stiffness and Metabolic Pathways

Systemic arterial stiffness, which reflects the combined stiffness of both central and peripheral arteries, is a key risk factor for CVD, renal dysfunction, and mortality [84]. It is commonly assessed using metrics such as the CAVI and baPWV, both of which have been widely used in clinical practice [85]. Notably, young men exhibit significantly higher baPWV than young women (by 0.77 m/s) [73], predicting a higher risk of cardiovascular disease.

Four types of exercise, i.e., continuous aerobic exercise, resistance training, interval aerobic training, and stretching training, have been shown to positively influence systemic arterial stiffness in young men (Table 4). Continuous aerobic exercise has consistently demonstrated reductions in systemic arterial stiffness. This includes light-intensity (30% HRR) [50], moderate-intensity cycling (35% to 50% HRR) [41,45,46,47,49,51], and high-intensity (60% PPO) cycling. For resistance training, light-intensity (40% 1RM) has been shown to reduce systemic arterial stiffness [52,54]. However, moderate-intensity resistance training at 80% 1RM was associated with negative effects on systemic arterial stiffness [86]. Interval aerobic training also demonstrated positive effects. This included, (i) 35% HRR with 15-min bouts separated by a 20-min rest [45], (ii) 50% HRR with 20 and 60-min intervals [46], and shown by Zhou et al, who reported decreases in systemic arterial stiffness [47]. For stretching exercises, Yamato et al reported a significant reduction in baPWV following a regimen of maximal exertion, consisting of 30-s stretches with 10-s relaxations for 40 min [38].

**Figure 4:**
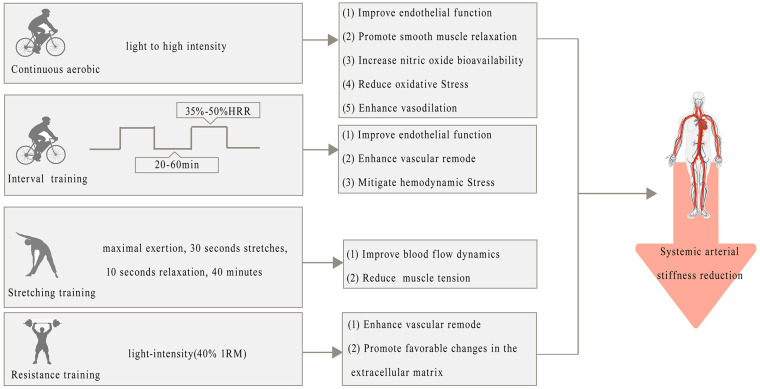
Positive effects of exercise on systemic arterial stiffness and metabolic pathways.

Exercise has a profound effect on systemic arterial stiffness, with various training modalities contributing to beneficial vascular adaptations. Continuous aerobic exercise, spanning intensities from light (30% HRR) to high (60% PPO), consistently reduces systemic arterial stiffness by improving endothelial function and promoting smooth muscle relaxation [87]. These effects are largely driven by increases in nitric oxide (NO) bioavailability, which enhances vasodilation and reduces oxidative stress [88]. Resistance training, particularly at light intensities (40% 1RM), also leads to a reduction in systemic arterial stiffness, potentially by enhancing vascular remodeling and promoting favorable changes in the extracellular matrix [89]. However, moderate-intensity resistance training (80% 1RM) has been shown to increase arterial stiffness, possibly due to the acute increases in blood pressure and sympathetic nervous system activity that may outweigh the benefits of vascular adaptations [63]. Interval aerobic training, especially at moderate intensities (35–50% HRR), also demonstrates reductions in arterial stiffness, likely to be due to the balance between hemodynamic stress and recovery, which promotes beneficial vascular remodeling and improved endothelial function [90]. Stretching exercises, although less extensively studied, have shown potential in reducing baPWV, possibly through the reduction in muscle tension and improved blood flow dynamics [91]. The underlying metabolic pathways involve the attenuation of oxidative stress, the reduction in systemic inflammation, and improvements in the function of the renin-angiotensin-aldosterone system, which collectively contribute to a more flexible and resilient arterial system. Over time, chronic exercise-induced reductions in systemic arterial stiffness are likely to involve long-term structural changes, including the preservation of elastin and reduction in collagen accumulation in arterial walls, which is essential for maintaining vascular elasticity and mitigating the risk of CVD (Figure 4).

### 3.5. Primary Exercise Recommendations

The main findings of this review are as follows: (i) For peripheral arterial stiffness, exercise modalities, such as continuous aerobic, interval aerobic, and stretching training, effectively reduce stiffness in young men. Continuous and interval aerobic cycling, particularly at moderate to high intensities, improves short-term vascular function by increasing nitric oxide availability and reducing sympathetic nervous system overactivation. Stretching training, when performed to the point of minimal discomfort, appears to benefit short-term vascular function through stress relief mechanisms. (ii) For central arterial stiffness, continuous and interval aerobic training, particularly at moderate intensities and over extended durations, are effective in achieving long-term reductions in stiffness. These improvements are primarily attributed to structural vascular adaptations, including elastin preservation and collagen regulation. (iii) For systemic arterial stiffness, exercise comprising continuous aerobic activity at moderate to high intensities, resistance with light-intensity, interval aerobic activity, and stretching training has a positive impact by enhancing endothelial function, promoting smooth muscle relaxation and improving vascular remodeling in young men.

### 3.6. Limitations and Directions for Further Research

Only a limited number of studies (three papers) have examined the effects of long-term exercise on arterial stiffness in young men. In addition, the precise mechanisms underlying these changes, including the roles of oxidative stress, nitric oxide, and autonomic regulation, remain inadequately explored and warrant further investigation to optimize exercise prescriptions. Future research should focus on the metabolic pathways underlying these vascular adaptations and examine how they differ across various populations.

## 4. Conclusions

A comprehensive exercise guideline for young men aiming to reduce peripheral, central, and systemic arterial stiffness should incorporate a combination of continuous aerobic, interval aerobic, resistance, and stretching exercises. For peripheral arterial stiffness, moderate- to high-intensity continuous and interval aerobic cycling is recommended, as these interventions enhance nitric oxide (NO) bioavailability and reduce sympathetic nervous system overactivation, thereby improving short-term vascular function. Additionally, stretching exercises performed to the point of minimal discomfort can further support vascular health by alleviating mechanical stress. To address central arterial stiffness, high-intensity continuous aerobic and submaximal interval aerobic training for about 6 weeks has proven effective. These exercises promote structural adaptations, such as elastin preservation and collagen regulation, which are essential for maintaining arterial elasticity. For systemic arterial stiffness, a diverse exercise regimen that includes light to high-intensity continuous aerobic exercises, light-intensity resistance training, and interval aerobic and stretching exercises is recommended. These modalities collectively improve endothelial function, promote smooth muscle relaxation, and facilitate vascular remodeling. By incorporating these tailored strategies, young men can effectively target arterial stiffness across different vascular regions and reduce their risk of cardiovascular disease.

## 5. Implications for Clinical Practice and Public Health

Given the higher prevalence of arterial stiffness and its link to increased cardiovascular risk in young men, early intervention through exercise is crucial. Exercise programs targeting peripheral, central, and systemic arterial stiffness, while simultaneously addressing metabolic dysfunction, could be key to preventing cardiovascular disease within this population. Public health initiatives that promote physical activity and educate young men about its benefits may help delay the onset of arterial stiffness and reduce long-term healthcare costs. Additionally, a deeper understanding of the metabolic mechanisms underlying arterial stiffness in these regions could facilitate the development of more targeted and effective interventions, ultimately improving cardiovascular health outcomes.

## Figures and Tables

**Table 1:** Web of Science search flow.

Filter	(English) and (In Title and Abstract and Keywords)	Results
#1	(exercise) and (young) and (men) and (arterial stiffness)	268
#2	(exercise) and (young) and (male) and (arterial stiffness)	127
#3	(exercise) and (young) and (men) and (PWV)	58
#4	(exercise) and (young) and (male) and (PWV)	22
#5	Total after merging duplicate articles from #1 to #4	314

**Table 2:** Suggested exercise interventions for peripheral arterial stiffness in young men (n = 11).

Author	Type	Time/Duration	Intensity	Main Finding
Jun Sugawara et al., 2003 [30]	Single-leg (left) cycling exercise	5 min	light-intensity (20 or 30 W) HR: 82 ± 2 bpm	faPWV (Peripheral) decreased in the exercised leg, but not in the counterpart
Kevin S. Heffernan et al., 2007 [31]	Cycling exercise		high-intensity Maximal aerobic capacity	Peripheral PWV decreased and was not recovered in 30 min
Katharine D. Currie et al., 2009 [32]	Cycling short-term endurance exercise	6 days, 120 min/day	high-intensity 65% VO_2_peak	Peripheral PWV decreased
Gerasimos Siasos et al., 2016 [33]	Acute aerobic cycling exercise	Continuous aerobic activity: 30 min; Interval aerobic activity: 30 bouts of 30 s, passive rest period of 30 s	Continuous aerobic activity: moderate-intensity 50% of the maximum aerobic work capacity Interval aerobic activity: high-intensity 100% maximum aerobic work capacity	fdPWV (Peripheral) decreased in both exercise types
Sun et al., 2020 [34]	Acute treadmill exercise	45 min	high-intensity 70% HRR	faPWV (Peripheral) decreased
Mark Rakobowchuk et al., 2009 [35]	Acute sprint interval exercise	(i) Single Wingate test, and (ii) 4 Wingate tests interspersed with 4.5 min recovery	High-intensity Wingate	Single Wingate: pPWV (Peripheral) decreased 4 Wingate: pPWV (Peripheral) decreased
Nicolas Tordi et al., 2010 [36]	Acute cycling exercise	6 consecutive periods of 5 min (a base of 4 min duration and a peak of 1 min duration)	moderate-intensity HR: 144.2 ± 2.4 bpm	llPWV (Peripheral) decreased
Ryota Kobayashi et al., 2018 [37]	Acute accumulated cycling exercise	Two 15 min bouts separated by 20 min rest	high-intensity 65% PPO	faPWV (Peripheral) decreased
Yosuke Yamato et al., 2016 [38]	Acute systemic static stretching exercise	40 min (30 s stretch, 10 s relaxation, 3 repetitions)	Maximal exertion	faPWV (Peripheral) decreased and returned to the baseline in 60 min
Yosuke Yamato et al., 2017 [39]	Acute stretching of one leg	6 repetitions of 30 s static stretch with a 10 s recovery		faPWV (Peripheral) decreased
Yuya Higaki et al., 2021 [40]	Acute stretching of one leg	30 s, 6 sets	Point of minimal discomfort	faPWV (Peripheral) decreased

**Table 3:** Suggested exercise interventions for central arterial stiffness in young men (n = 6).

	Type	Time/Duration	Intensity	Main Finding
Katharine D. Currie et al., 2009 [32]	Cycling short-term endurance exercise	6 days, 120 min/day	high-intensity 65%VO_2_peak	Central PWV decrease
Sophy J Perdomo et al., 2016 [41]	Acute treadmill exercise	30 min	high-intensity 73.0 ± 4.9% HRmax	cfPWV (Central) decreased
Natsuki Hasegawa et al., 2018 [42]	Cycling exercise	Continuous aerobic activity: 8 weeks 3 days/week, 45 min Interval aerobic activity: 4 days/week, 6–7 sets of 20 s exercise with 10 s rest between sets	Continuous aerobic activity: moderate-intensity 60–70% VO_2_max Interval aerobic activity: high-intensity 170% of VO_2_max	cfPWV (Central) decreased
Matthew Cocks et al., 2013 [43]	6 weeks treadmill exercise	Continuous aerobic activity: 40–60 min, 5 times/week; Interval aerobic activity: 3 times a week, 4–6 Wingate tests, interspersed with 4.5 min recovery	high-intensity Continuous aerobic activity: 65% VO_2_peak; Interval aerobic activity: Wingate	Central PWV decreased
Ryota Kobayashi et al., 2018 [37]	Acute accumulated cycling exercise	2 bouts of 15 min cycling separated by 20 min intervals	high-intensity 65% PPO	cfPWV (Central) decreased
Ryan M. Sapp et al., 2020 [44]	Acute cycling exercise	Beginning with 6 min at 40% PPO followed by 3 min intervals at 85% PPO interspersed with 4 min intervals at 40% PPO.	high-intensity 60% PPO	cfPWV (Central) decreased

**Table 4:** Suggested exercise interventions for systemic arterial stiffness in young men (n = 11).

	Type	Time/Duration	Intensity	Main Finding
Hailin Wang et al., 2014 [45]	Acute cycling exercise	Continuous aerobic activity: 30 min; Interval aerobic activity: 2 15-min bouts separated by a 20-min rest	moderate-intensity 35% HRR	CAVI (Systemic) decreased in both continuous and interval aerobic exercise
Lu Zheng et al., 2015 [46]	Acute cycling exercise	Continuous aerobic activity: 30 min; Interval aerobic activity (20): 2 × 15-min separated by a 20-min rest; Interval aerobic activity (60): 2 × 15-min separated by a 60-min rest	moderate-intensity 50% HRR	CAVI (Systemic) decreased in all exercise types
Zhixiong Zhou et al., 2015 [47]	Acute cycling exercise	Continuous aerobic activity: 30 min; Interval aerobic activity(10): 10 min aerobic, 3 repeats, 10 min interval Interval aerobic activity (60): 10 min aerobic, 3 repeats, 60 min interval	moderate-intensity 50% HRR	CAVI (Systemic) decreased in all exercise types
Takanobu Okamoto et al., 2019 [48]	Acute eccentric versus concentric cycling	First concentric: 30 min Second concentric: 30 min First eccentric: 30 min Second eccentric: 30 min	high-intensity 60% PPO	baPWV (Systemic) Decreased was shown in the first concentric only at 0.5 h; no change in the second concentric, first eccentric and second eccentric
Jun Yin et al., 2019 [49]	Acute cycling exercise	Cycling at 60 rounds per min trial (RPM 60): 30 min Cycling at 90 rounds per minute trial (RPM 90): 30 min	moderate-intensity 35% HRR	CAVI (Systemic) RPM 60: decreased, RPM 90: decreased
Qi Huang et al., 2021 [50]	Acute aerobic exercise	Cycling program (CYC): 30 min Zero incline running Program (TM0): 30 min 18% incline running Program (TM18): 30 min	moderate -intensity 35% HRR	CAVI (Systemic) CYC: decreased TM0: decreased TM18: decreased
Zhixiong Zhou et al., 2022 [51]	Acute cycling exercise	Continuous aerobic activity: 30 min; Interval aerobic activity of long bouts with long intervals: 10 min cycling, 20 min interval; Interval aerobic activity of long bouts with short intervals: 10 min cycling, 5 min interval; Interval aerobic activity of short bouts with short interval: 5 min cycling, 15 min interval	moderate-intensity 35% HRR	CAVI (Systemic) decreased in all exercise types
Arturo Figueroa et al., 2011 [52]	Acute squat with WBV	10 sets of 1 min static squat separated by 1 min rest between sets, knees flexed at 120°	light-intensity HR: 69 ± 4 bpm	baPWV (Systemic) decreased
Tianjiao Wang et al., 2024 [53]	Acute High intensity intermittent training(HIIT)	Cycling-type high intensity interval training (C-HIIT): 10 × 60 s separated by 60 s active recovery; Resistance-type high intensity interval training (R-HIIT): 10 × 60 s separated by 60 s passive recovery	high-intensity C-HIIT: cycling, 90%PPO separated by 25%PPO; R-HIIT: squat with a load of 20% bodyweight, maximum 30 reps separated by passive recovery position	CAVI (Systemic) C-HIIT: decreased R-HIIT: decreased
Yosuke Yamato et al., 2016 [38]	Acute systemic static stretching exercise	40 min (30 s stretch, 10 s relaxation, 3 repetitions)	high-intensity Maximal exertion	baPWV (Systemic) decreased, returned to the baseline in 60 min.
Chongwen Zuo et al., 2022 [54]	6 weeks’ resistance exercise	3 times/week Traditional resistance training (TRT): 4–5 sets, 8–12 reps, rest 1–2 min between sets Functional resistance training(FRT): 4–5 sets, 15–22 reps, rest 1–2 min between sets, and used BOSU ball, swish balls, and balance discs	moderate-intensity TRT: 70% 1RM FRT: 40% 1RM	CAVI (Systemic) TRT: decreased FRT: decreased

## Data Availability

Not applicable.

## Abbreviations

The following abbreviations are used in this manuscript:

CVD	Cardiovascular diseases
faPWV	foot-to-brachial pulse wave velocity
cfPWV	carotid–femoral pulse wave velocity
CAVI	cardio-ankle vascular index
PAD	peripheral artery disease
pPWV	peripheral pulse wave velocity
fdPWV	femoral–distal pulse wave velocity
ulPWV	upper limb pulse wave velocity
brPWV	brachial artery pulse wave velocity
bPWV	brachial pulse wave velocity
llPWV	lower limb pulse wave velocity
cPWV	central pulse wave velocity
aPWV	aortic pulse wave velocity
baPWV	brachial–ankle pulse wave velocity
HR	heart rate
HRR	heart rate reserve
AGEs	advanced glycation end-products
NO	nitric oxide
HRmax	Maximum Heart Rate
VO_2_peak	Peak oxygen uptake
VO_2_max	Maximum Oxygen Consumption
RM	Repetition Maximum
PPO	Peak Power Output

## Appendix A. Critical Appraisal Using the Nine Item Checklist by Hawker et al.

**Table A1:** Quality appraisals of peripheral arterial stiffness. Good = 4 points, Fair = 3 points, Poor = 2 points, Very poor = 1 point.

Study	1. Title and Abstract	2. Introduction and Aims	3. Method and Data	4. Sampling	5. Data Analysis	6. Ethics and Bias	7. Results	8. Transferability and Generalizability	9. Implications and Usefulness	Total Score (max: 33)
Jun Sugawara et al., 2003 [30]	3	4	3	3	4	3	4	3	3	30
Kevin S. Heffernan et al., 2007 [31]	4	3	2	4	3	4	3	4	3	30
Katharine D et al., 2009 [32]	3	4	3	3	3	4	3	3	3	29
Gerasimos Siasos et al., 2016 [33]	4	3	3	3	4	3	4	3	4	31
P. Sun et al., 2020 [34]	3	3	3	4	3	4	4	4	4	32
Mark Rakobowchuk et al., 2009 [35]	3	4	3	2	4	3	3	2	4	28
Nicolas Tordi et al., 2010 [36]	3	3	4	3	3	3	4	3	4	30
Ryota Kobayashi et al., 2018 [37]	3	4	3	2	4	3	3	2	3	27
Yosuke Yamato et al., 2016 [38]	3	4	3	4	4	3	4	4	4	33
Yosuke Yamato et al., 2017 [39]	4	3	2	3	4	4	3	3	3	29
Yuya Higaki et al., 2021 [40]	3	4	3	3	3	3	3	4	4	30
Mean ± SD	3.3 ± 0.5	3.5 ± 0.5	2.9 ± 0.5	3.1 ± 0.7	3.5 ± 0.5	3.4 ± 0.5	3.5 ± 0.5	3.2 ± 0.8	3.3 ± 0.5	39.9 ± 1.7

**Table A2:** Quality appraisals of central arterial stiffness. Good = 4 points, Fair = 3 points, Poor = 2 points, Very poor = 1 point.

Study	1. Title and Abstract	2. Introduction and Aims	3. Method and Data	4. Sampling	5. Data Analysis	6. Ethics and Bias	7. Results	8. Transferability and Generalizability	9. Implications and Usefulness	Total Score (max: 32)
Katharine D et al., 2009 [32]	3	4	3	3	3	4	3	3	3	29
Sophy J Perdomo et al., 2016 [41]	3	3	3	3	4	4	4	3	3	30
Natsuki Hasegawa et al., 2018 [42]	3	4	4	3	3	4	4	3	4	32
Matthew Cocks et al., 2013 [43]	4	3	4	3	3	3	3	4	3	30
Ryota Kobayashi et al., 2018 [37]	3	4	3	2	4	3	3	2	3	27
Ryan M. Sapp et al., 2020 [41]	3	4	3	3	3	3	4	3	4	30
Mean ± SD	3.2 ± 0.4	3.7 ± 0.5	3.3 ± 0.5	2.8 ± 0.4	3.3 ± 0.5	3.5 ± 0.5	3.5 ± 0.5	3.0 ± 0.6	3.3 ± 0.5	29.7 ± 1.6

**Table A3:** Quality appraisals of systemic arterial stiffness. Good = 4 points, Fair = 3 points, Poor = 2 points, Very poor = 1 point.

Study	1. Title and Abstract	2. Introduction and Aims	3. Method and Data	4. Sampling	5. Data Analysis	6. Ethics and Bias	7. Results	8. Transferability and Generalizability	9. Implications and Usefulness	Total Score (max: 33)
Hailin Wang et al., 2014 [45]	4	3	3	3	4	3	4	3	4	31
Lu Zheng et al., 2015 [46]	3	4	3	3	3	3	3	3	3	28
Zhixiong Zhou et al., 2015 [47]	3	3	4	3	3	4	3	4	3	30
Takanou Okamoto et al., 2019 [48]	3	4	3	3	4	4	3	3	3	30
Jun Yin et al., 2018 [49]	3	4	3	3	3	3	3	3	3	28
Qi Huang et al., 2021 [50]	3	4	3	3	3	4	4	3	4	31
Zhixiong Zhou et al., 2022 [51]	4	3	3	3	4	3	3	3	3	29
Arturo Figueroa et al., 2011 [52]	3	4	3	3	3	3	4	3	4	30
Tianjiao Wang et al., 2024 [53]	4	3	3	3	4	3	4	3	4	31
Yosuke Yamato et al., 2016 [38]	3	4	3	4	4	3	4	4	4	33
Chongwen Zuo et al., 2022 [54]	3	4	4	4	4	3	3	4	3	32
Mean ± SD	3.3 ± 0.5	3.6 ± 0.5	3.2 ± 0.4	3.2 ± 0.4	3.6 ± 0.5	3.3 ± 0.5	3.5 ± 0.5	3.3 ± 0.5	3.4 ± 0.5	30.3 ± 1.6
